# Socioeconomic Position and Low Birth Weight among Mothers Exposed to Traffic-Related Air Pollution

**DOI:** 10.1371/journal.pone.0113900

**Published:** 2014-11-26

**Authors:** Mateus Habermann, Nelson Gouveia

**Affiliations:** Department of Preventive Medicine, School of Medicine, University of São Paulo, São Paulo, Brazil; University of Missouri, United States of America

## Abstract

**Background:**

Atmospheric pollution is a major public health concern. It can affect placental function and restricts fetal growth. However, scientific knowledge remains too limited to make inferences regarding causal associations between maternal exposure to air pollution and adverse effects on pregnancy. This study evaluated the association between low birth weight (LBW) and maternal exposure during pregnancy to traffic related air pollutants (TRAP) in São Paulo, Brazil.

**Methods and findings:**

Analysis included 5,772 cases of term-LBW (<2,500 g) and 5,814 controls matched by sex and month of birth selected from the birth registration system. Mothers’ addresses were geocoded to estimate exposure according to 3 indicators: distance from home to heavy traffic roads, distance-weighted traffic density (DWTD) and levels of particulate matter ≤10 µg/m^3^ estimated through land use regression (LUR-PM_10_). Final models were evaluated using multiple logistic regression adjusting for birth, maternal and pregnancy characteristics. We found decreased odds in the risk of LBW associated with DWTD and LUR-PM_10_ in the highest quartiles of exposure with a significant linear trend of decrease in risk. The analysis with distance from heavy traffic roads was less consistent. It was also observed that mothers with higher education and neighborhood-level income were potentially more exposed to TRAP.

**Conclusions:**

This study found an unexpected decreased risk of LBW associated with traffic related air pollution. Mothers with advantaged socioeconomic position (SEP) although residing in areas of higher vehicular traffic might not in fact be more expose to air pollution. It can also be that the protection against LBW arising from a better SEP is stronger than the effect of exposure to air pollution, and this exposure may not be sufficient to increase the risk of LBW for these mothers.

## Introduction

Interest in pregnancy outcomes has been increasing within the field of environmental epidemiology over the last few years. Pregnancy constitutes a period of high susceptibility to environmental pollution and its different adverse outcomes are important indicators of the potential impacts of pollution on child health. For example, prematurity [1], [2], [3], [4] and growth restriction [1], [2], [3], [5], [6], [7] are some of the outcomes that have been examined in relation to ambient air pollution. Among these, great attention has been given to low birth weight (LBW) due to its relative higher frequency and because LBW is considered to be a good indicator of newborns’ health status and survival. Moreover, it is a condition that is associated with outcomes in adulthood [8], [9], [10].

Epidemiological studies on the association between air pollution and LBW have been providing conflicting results. Some studies have found an increased risk of LBW associated to greater exposure to pollutants [6], [7], [11], [12], [13], while others found inconsistent or no associations [2], [3], [14]. There are also studies that found an inverse relationship between exposure to ambient air pollution and LBW [3], [7], [15], [16]. Nonetheless, recent review articles and meta-analysis studies [17], [18], [19] have suggested that associations likely do exist, especially for exposure to airborne particles (PM_2.5_ and PM_10_).

The divergence observed among studies is probably due to differences in the approaches taken towards assessing the population exposure, types of sources and pollutants in different locations [16], [20], [21], different populations’ characteristics (ethnicity, social situation and prevalence of maternal smoking) [10] or it might also be the result of some unmeasured factor. Given these potential problems, further studies are needed to confirm that the effect of air pollution on birth weight is indeed causal [10].

Many of these studies have evaluated the exposure to atmospheric air pollution using the mean concentration of pollutants measured by air quality monitoring stations. Although this approach allows the evaluation of specific air pollutants separately and with temporal accuracy some argue that monitoring stations are sparse and not sited everywhere people live [21]_._ For economic and administrative reasons there are limitations in the number and distribution of these stations and, thus, they do not detect precisely the spatial heterogeneity of pollutant concentration [13], [21], [22].

Moreover, there are locations at which levels of air pollution are higher due to greater emissions by heavy traffic or in the proximity of industrial plants, railway stations, airports and ports or due to limitations on air pollution dispersion, such as in street canyons [23]. Therefore, if the information used to assess population exposure only comes from monitoring stations, it might not be possible to identify a specific source of pollution to support decision policy makers in designing effective regulation.

Consequently, recent investigations have applied exposure assessment methods based on emission sources using information from roads and traffic. Indicators frequently seen in the literature include the shortest distance from points of interest to roads with heavy traffic flows – highways or main roads [1], [2], [4] and the distance-weighted traffic density (DWTD) [5], [7], [24]. Land use regression (LUR) is another method that has become frequent in studies of the health effects of atmospheric pollution [14], [22]. This method accurately detects spatial heterogeneity of pollutants improving the assessment of exposure [25].

Confounding by socioeconomic position (SEP) in the association between air pollution and adverse pregnancy outcomes is also of concern. Some studies suggest that lower SEP population are exposed to higher levels of air pollution [26], [27], [28], while the newborn health itself is associated to low SEP [5], [24]. Furthermore people with lower SEP are more likely to accumulate environmental exposure from multiple sources e.g. noise, water quality, crowding, housing quality, smoking, nutritional status, criminality and domestic violence [1], [26].

São Paulo has one the largest vehicle fleets in the world circulating in approximately 16.300 km of roads in a densely populated area. Vehicle traffic emissions are the major contributor towards high levels of atmospheric pollution [29]. The city is very socially unequal and segregated, thus the way the population is spatially distributed implicates in heterogeneity of exposure to air pollution among socioeconomic groups.

Therefore, the aim of the present study was to examine the association between LBW and exposure of mothers to traffic related air pollution (TRAP) during pregnancy using three different indicators of exposure based on vehicle traffic. In addition, we evaluated the role of socioeconomic position in this association.

## Methods

### 1. Ethics Statement

This study was approved by the Research Ethics Committee of the School of Medicine, University of São Paulo (Research Protocol 0669/09). The individual maternal and newborn characteristics, pregnancy and delivery information of the subjects were obtained from the birth certificate records and they were anonymized before the analysis.

### 2. Subjects

This case-control study was conducted in São Paulo, the largest city in Brazil with 11.446.275 inhabitants. The population of the study comprised all live births to mothers living in the municipality of São Paulo in 2006 that were registered in the Brazilian Live Birth Information System (SINASC) (n = 173,566). This system has very high population coverage for São Paulo (greater than 99%) [30].

Single births, at hospital environment, at full term (37–41 weeks of gestation) and with a birth weight ≥1,000 g and <5,500 g were included in the study. Birth records without address information were excluded (n = 6,401; 3.7%). After this first selection, a total of 145,724 births (83.9%) were considered in the study.

Cases were defined as full term live births with a weight <2,500 g. All LBW children (n = 5,985, 4.2%) were included in the study and frequency matched to a random selection of controls according to sex and month of birth at a ratio 1∶1. The residential address of mothers of each case and control was geocoded using Mapinfo software (Professional version 8.5; MapInfo Corporation, New York, NY, USA).

To further evaluate the potential confounding by socioeconomic position (SEP) beyond maternal education (available in the birth certificate), we obtained information on income of the head-of-household at the census tract level from the 2000 population Census (1 minimum wage R$ 151 or US$ 85). We then assigned each mother the average level of income observed in the census tract where her residence was contained.

### 3. Road and traffic data

For this study, road and vehicle traffic information from the year 2007 was used. These data included the cartographic base exhibiting streets classified according to the number of lanes of traffic and the maximum permitted speed in each segment. The Traffic Engineering Company (CET) of São Paulo performs a traffic counting process at 32 selected roads and uses the software EMME-2 to simulate traffic (vehicles/hour) in collector, arterial and rapid transit roads for the whole city. This method is not applied to local roads.

The city of São Paulo has more than 16,300 km of roads and almost 75% of them are classified as local. Less than 2% are rapid transit roads, with greatest traffic flows.

Traffic in local roads was estimated by CET in 926 demarcated regions for traffic and public transport planning (origin-destination zones). The sum of the traffic in each of these regions was divided by the sum of the extent of local streets contained in each region. From this, the traffic density was obtained in terms of the number of vehicles per meter of local street, in each region (*i*). This measurement was then multiplied by the length of the segments of local roads (*SLR*), in meters, contained in the respective regions, thereby obtaining the volume of traffic on each stretch of the local streets (*T*).
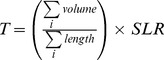
(1)


### 4. Evaluation of the exposure

Maternal residential exposure to atmospheric air pollution was evaluated through three approaches commonly encountered in the literature: the shortest distance from home to roads with heavy traffic, the DWTD and LUR. All three indicators were constructed using the annual average of traffic counts according to road type in the city of Sao Paulo in 2007 obtained from the Traffic Engineering Company of the city of Sao Paulo (CET-SP).

### 5. Distance from roads with heavy traffic flows

We defined roads with heavy traffic flow those with vehicle traffic volumes greater than the 95^th^ percentile (≥1,876 vehicles/hour) of the distribution. This included most of the rapid transit, arterial and collector roads. The shortest distance between the mother’s home address and these roads were then estimated.

### 6. Distance-weighted traffic density

In the DWTD indicator, it is assumed that the dispersion of emissions produced by vehicles on roads approximates to a Gaussian (normal) distribution, and that 96% of the pollutants spreads within a distance of up to 500 feet (150 m) from the center of the road as the model developed and applied by Pearson et al. (2000) [31].

For each subject studied, the shortest distances to roads within a radius of 750 feet (228.6 m) around the mother’s residential address was calculated. For each distance (*D*), the value *Y* was calculated as a weighting factor for vehicle flows obtained for each road within the area.
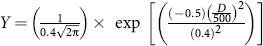
(2)


The *Y* was used to weigh the products of the traffic intensities of all road segments within the buffer. The weighted values were summed for each subject to obtain the DWTD.

### 7. Land use regression

A previous LUR model developed for the city of Sao Paulo was used to provide estimated local levels of air pollution [32]. Briefly, mean concentrations of PM_10_ from the monitoring network were obtained for the year 2007 at 09 sites in the study area.

For each of the measurement sites 113 variables of land use (residential, commercial, industrial, public areas, open areas, mixed areas etc.), road type (local, collector, arterial and fast transit) and traffic (average of vehicles/hour by road segment) were generated in a geographic information system (GIS) (Mapinfo Professional version 8.5; Mapinfo Corporation, New York, NY, USA) in buffer zones of 250 to 1,000 m of radius. Univariate regression models were built with these covariates to identify the variables mostly related to PM_10_ levels. The variables that exhibited more robust associations were related to light traffic, number of households, commercial/services/industrial land use (areas with 3 mixed predominant land uses) and residential land use. The final model had an *R* ^2^ of 0.638 and included light traffic within 250 m radius circular buffers of the measurement sites.

We then computed individual maternal exposure to PM_10_ applying the model at mothers’ homes addresses.

### 8. Statistical analysis

First, univariate logistic regression was performed on each variable indicative of maternal, newborn, pregnancy and delivery characteristics, so that possible confounding factors could be identified. The association measurement used was the odds ratios (OR), with 95% confidence intervals and considering a 5% significance level (α = 5%).

We adjusted the multivariate model for several recognized predictors of reduced birth weight that could potentially confound the relationship between LBW and the indicators of exposure [5], [11], [16], [24]. We kept these variables in the final models if they attained statistical significance (*p*<0.05) in the likelihood ratio test with a saturated model or if we considered them important potential confounders of the association between our indicator of exposure and LBW, regardless of their significance.

The exposure to vehicle traffic indicated by the distance to roads ≥95^th^ percentile of traffic was tested in quartiles and dichotomized as <150 m and ≥150 m since air dispersion models suggest that most constituents of automotive exhaust decrease to background concentrations within this distance [31], [33]. The DWTD and the estimated PM_10_ based on LUR (LUR-PM_10_) were also examined in quartiles.

All the statistical analysis was conducted using the SPSS for Windows software (SPSS Inc., Chicago, IL, USA).

## Results

Of the 11,970 subjects selected for this study, we were unable to geocode the residential address in only 3.2% (213 cases and 171 controls). Therefore, 11,586 subjects remained in the analysis: 5,772 cases and 5,814 controls. Subjects not included had similar characteristics to those analyzed except that they had less educated mothers and a higher proportion of mixed race (data not showed).

We calculated non parametric Spearman test to verify the correlation among the metrics of exposure. The correlation were stronger between LUR-PM_10_ and DWTD (r = 0.772) and also between LUR-PM_10_ and distance to roads ≥95^th^ percentile of traffic (r = −0.623). The correlation between DWTD and distance was moderate (r = −0.508). All correlations were statistically significant (p<0.001).

Controls lived closer than cases to busy roads (p = 0.003). The levels of LUR-PM_10_ were also higher for controls compared to cases (p = 0.04). This scenario indicates controls had higher average exposure to TRAP than cases (Table 1).

**Table 1:** Descriptive statistic of the exposure variables shortest distance to roads ≥95th percentile of traffic, DWTD and LUR-PM10 between cases of LBW and controls.

		n	Minimum	Mean (SD)	Median	25^th^Percentile	75^th^ Percentile	Máximum
**DWTD (vehicles/hour)**	**All**	*11,586*	*0.007*	*599.7 (±1,010.8)*	*188.7*	*22.5*	*763.6*	*10,331.1*
	**Controls**	*5,814*	*0,012*	*615.3 (±1,003.1)*	*209.6*	*25.1*	*800.4*	*10,331.1*
	**Cases**	*5,772*	*0.012*	*583.9 (±1,018.3)*	*168.8*	*20.8*	*730.0*	*10,331.1*
**Distances (meters)** § *	**All**	*11,586*	*0*	*956.2 (±1,323.2)*	*547.0*	*249.4*	*1,126.4*	*22,263.7*
	**Controls**	*5,814*	*0*	*919.7 (±1,246.3)*	*521.7*	*241.4*	*1,090.5*	*17063.9*
	**Cases**	*5,772*	*0.11*	*993.0 (±1,395.5)*	*564.9*	*258.1*	*1,159.3*	*22,263.7*
LUR-PM_10_ (µg/m^3^)**	**All**	*11,586*	*35.3*	*39.1 (±6.0)*	*37,0*	*35.3*	*40.4*	*108.2*
	**Controls**	*5,814*	*35,3*	*39.2 (±5.9)*	*37.1*	*35.3*	*40.7*	*108.2*
	**Cases**	*5,772*	*35.1*	*39.0 (±6.1)*	*36.8*	*35.3*	*40.1*	*108.2*

§ Shortest distance to roads ≥95^th^ percentile of traffic.

Statistical significance of the difference between cases and controls (*p = 0.003, **p = 0.041).

Cases and controls were not equally distributed according to sociodemographic and other characteristics, as shown in Table 2. As expected, compared to controls mothers of LBW children had less antenatal care visits.

**Table 2:** Subjects [no. (%)] and univariate ORs according to socioeconomic, demographic, maternal and fetal characteristics, and the exposure to the three indicators of air pollution between cases of LBW and controls.

Antenatal care**	Cases (%)		Controls (%)		OR (95%CI)
No visits	*111 (1.9%)*		*53 (0.9%)*		*2.35 (1.69; 3.27)*
1 to 3	*359 (6.4%)*		*226 (3.9%)*		*1.78 (1.50; 2.12)*
4 to 6	*1398 (24.7%)*		*1207 (21.0%)*		*1.30 (1.19; 1.42)*
≥7	*3795 (67.0%)*		*4263 (74.2%)*		*1.0*
Maternal education**					
≤3 years	*271 (4.8%)*		*240 (4.2%)*		*1.39(1.14; 1.68)*
4 to7 years	*1375 (24.1%)*		*1142 (19.8%)*		*1.48 (1.31; 1.66)*
8 to 12 years	*3075 (54.0%)*		*3180 (55.3%)*		*1.18 (1.07; 1.31)*
>12 years	*974 (17.1%)*		*1194 (20.7%)*		*1.0*
Number of previous births**					
No child	*3151 (54.6%)*		*2767 (47.6%)*		*1.36 (1.26; 1.47)*
1 to 3	*2331 (40.3%)*		*2792 (47.9%)*		*1.0*
≥4	*290 (5.0%)*		*255 (4.4%)*		*1.36 (1.14; 1.63)*
Marital status**					
Single	*3644 (63.7%)*		*3412 (59.0%)*		*1.26 (1.16; 1.36)*
Married	*1913 (33.4%)*		*2267 (38.9%)*		*1.0*
Widow	*13 (0.2%)*		*11 (0.2%)*		*1.39 (0.62; 3.11)*
Separed/divorced	*66 (1.2%)*		*65 (1.1%)*		*1.19 (0.84; 1.69)*
Consensual union	*87 (1.5%)*		*41 (0.7%)*		*2.47 (1.69;3.60)*
Maternal age**					
<20	*1009 (17.5%)*		*812 (14.0%)*		*1.32 (1.19; 1.47)*
20 to 29	*2831 (49.0%)*		*3004 (51.7%)*		*1.0*
30 to 39	*1736 (30.1%)*		*1822 (31.3%)*		*1.01 (0.93; 1.10)*
≥40	*196 (3.4%)*		*176 (3.0%)*		*1.18 (0.96; 1.46)*
Number of previous stillbirths**					
No child	*5283 (91.5%)*		*5433 (93.4%)*		*1.0*
≥1	*489 (8.5%)*		*381 (6.6%)*		*1.32 (1.15; 1.51)*
**Delivery**					
Vaginal	*2814 (48.8%)*		*2746 (47.3%)*		*1.0*
Cesarean	*2951 (51.2%)*		*3065 (52.7%)*		*0.94 (0.88; 1.01)*
**Neighborhood-level income** † **					
<3.35	*1495 (25.9%)*		*1393 (23.7%)*		*1.25 (1.13; 1.39)*
3.35 to <4.62	*1500 (26.0%)*		*1412 (24.3%)*		*1.22 (1.10; 1.35)*
4.62 to 7.16	*1423 (24.7%)*		*1472 (25.3%)*		*0.98 (0.98; 1.23)*
≥7.16	*1350 (23.4%)*		*1553 (26.7%)*		*1.0*
Race/ethnicity**					
White	*3217 (66.4%)*		*3397 (70.2%)*		*1.0*
Black	*110 (2.3%)*		*73 (1.5%)*		*1.59 (1.18; 2.15)*
Asian	*19(0.4%)*		*29 (0.6%)*		*0.7 (0.4; 1.24)*
Mixed races	*1501 (30.9%)*		*1343 (27.7%)*		*1.18 (1.08; 1.29)*
**Gender**					
Male	*2444 (42.0%)*		*2425 (42.0%)*		*1.0*
Female	*3347 (58.0%)*		*3370 (58.0%)*		*1.00 (0.93; 1.08)*
LUR-PM_10_ (µg/m^3^)**					
<35.3	*1580 (27.4%)*		*1372 (23.9%)*		*1.0*
35.3 to <37.0	*1441 (24.5%)*		*1379 (23.7%)*		*0.92 (0.83; 1.02)*
37.0 to <40.4	*1404 (24.3%)*		*1493 (25.7%)*		*0.85 (0.76; 0.94)*
40.4 to ≤108.2	*1375 (23.8%)*		*1521 (26.2%)*		*0.81 (0.73; 0.90)*
DWTD (vehicles/hour)*					
<22.5	*1484 (26.1%)*		*1372 (23.9%)*		*1.0*
22.5 to <188.7	*1441 (25.4%)*		*1415 (24.7%)*		*0.94 (0.85; 1.04)*
188.7 to <763.6	*1392 (24.5%)*		*1464 (25.5%)*		*0.88 (0.79; 0.97)*
763.6 to ≤10,331.1	*1367 (24.0%)*		*1489 (25.9%)*		*0.85 (0.76; 0.94)*
**Distance (meters)** §					
<150	*844 (15.2%)*		*881 (14.6%)*		*0.96 (0.87; 1.06)*
≥150	*4928 (84.8%)*		*4933 (85.4%)*		*1.0*
**Distance (meters)** § *					
<249.4	*1394 (24.2%)*		*1501 (25.8%)*		*0.87 (0.78; 0.96)*
249.4 to <547.0	*1397 (24.2%)*		*1501 (25.8%)*		*0.87 (0.78;0.96)*
547.0 to <1,126.4	*1484 (25.7%)*		*1413 (24.3%)*		*0.98 (0.88; 1.01)*
1,126.4 to ≤22,263.7	*1497 (25.9%)*		*1399 (24.1%)*		*1.0*

χ^2^ test **p≤0.001, *p≤0.01.

† quartiles of minimum wages.

§ Shortest distance to roads ≥95^th^ percentile of traffic.

Cases and controls were not equally distributed according to sociodemographic and other characteristics, as shown in Table 2. As expected, the risk of having a LBW child was greater for mothers with no antenatal care visits (OR 2.35 [CI 95% 1.69; 3.27]), less education (OR 1.39 [CI 95% 1.14; 1.68]), younger (OR 1.32 [CI 95% 1.19; 1.47]), and living in neighborhoods of lower income [OR 1.25 (CI 95% 1.13; 1.39)]. In addition, compared to controls, mothers of LBW children were more likely to have previous stillbirths, being single or living in a consensual union and were of black or mixed races. The risk of LBW children was also greater among primiparous mothers (OR 1.36 [CI 95% 1.26; 1.47]) and for those with 4 or more previous births (OR 1.36 [CI 95% 1.14; 1.63]) compared to those with 1 to 3 previous births.

Most of these variables are also inter-related. For example, younger, single mothers, those with less education and living in poor neighborhoods exhibited significant less number of antenatal care visits (p<0.05).

In the univariate analysis we observed significant decreased odds for all 03 indicators of exposure to traffic-related air pollution: distance to busy roads (p = 0.006), DWTD (p = 0.010) and LUR-PM_10_ (p<0.001). Mothers with exposure in the highest quartiles of LUR-PM_10_, DWTD and distance had respectively 19%, 15% and 13% less risk of having a LBW child (Table 2).

The adjusted odds ratios (AOR) for DWTD and LUR-PM_10_ in the multivariate models maintained the decreased odds in the highest quartiles of exposure with a significant linear trend of decrease in risk. The results of the analysis using distance to heavy traffic roads also exhibited decreased odds, bus less consistent (Table 3). We also included in the final model the neighborhood-level income variable but results remained unchanged. Detailed information about the multivariate models is available in Tables S1, S2 and S3.

**Table 3:** Adjusted odds ratios (AOR) for LBW for each indicator of exposure in the multivariate models.

Distance (meters)§	AOR^a^ (95% CI)
<249.4	*0.93 (0.82; 1.4)*
249.4 to <547.0	*0.92 (0.82; 1.03)*
547.0 to <1,126.4	*1.00 (0.89; 1.11)*
1,126.4 to ≤22,263.7	*1.0*
**Distance (meters)** §	
<150	*0.97 (0.87; 1.08)*
≥150	*1.0*
DWTD (vehicles/hour)*	
<22.5	*1.0*
22.5 to <188.7	*0.96 (0.86; 1.07)*
188.7 to <763.6	*0.91 (0.82; 1.02)*
763.6 to ≤10,331.1	*0.90 (0.80; 1.01)*
LUR-PM_10_ (µg/m^3^)**	
<35.3	*1.0*
35.3 to <37.0	*0.93 (0.83; 1.03)*
37.0 to <40.4	*0.88 (0.79; 0.98)*
40.4 to ≤108.2	*0.86 (0.76; 0.96)*

a adjusted for antenatal care visits, number of previous alive births, number of previous stillbirths, maternal education, census-based income, marital status, maternal age and delivery.

§ Shortest distance to roads ≥95th percentile of traffic.

Statistical significance (*p<0.003, **p = 0.044).

We also analyzed in univariate and multivariate logistic regressions the exposure measures as continuous variables and the results also exhibited decreased odds. We also performed linear regression with birth weight as a continuous variable and results were similar (data not showed).

Finally, we examined the distribution of the three indicators of exposure according to levels of maternal education and the neighborhood-level income. Mothers more educated and residing in wealthier areas were significantly (p<0.001) more exposed to traffic-related air pollution. For more detail see Tables S4 and S5.

## Discussion

We evaluated the association between traffic-related air pollution and LBW using individual child, birth and maternal information and three different approaches to assess exposure. Unexpectedly, we observed significant decreased odds in risk of term LBW in infants born to mothers with higher exposure to traffic-related air pollution assessed by DWTD and LUR-PM_10_.

We could find only one study in the literature that obtained results similar to ours. Zeka et al. [15] found significant increase in birth weight associated with the shortest distance of mother's residence to major highways in Massachusetts, USA. Kashima et al. [16] in Japan did not find associations between traffic-based exposure (distance to major roads, LUR and DWTD) and LBW, although their effect estimates were in the same direction as ours for some of those indicators, i.e. mothers with higher exposure to NO_2_-LUR and living closer to major roads had less risk of having a LBW child. In the Netherlands associations were also not found between birth weight and maternal exposure to DWTD, distance to major roads [2] and LUR [3]. However, other studies have found an increased risk of LBW associated with higher exposure to air pollution either assessed by proximity to highway [1], DWTD [5] or by LUR [6].

Our results also disagree with previous studies conducted in Sao Paulo. Gouveia et al. [11] found a decrease in birth weight associated with higher maternal exposure to air pollution based on measurements from monitoring stations. Another study [24] suggested a gradient of increasing risk of early neonatal death with higher exposure to traffic-related air pollution. Mothers exposed to the highest quartile of the DWTD exhibited approximately 50% increased risk.

The difference between our results and those conducted in other locations but using similar methodology might be at least partially explained by differences in population characteristics, underlying patterns of morbidity or in the mixture and composition of air pollutants. With regard to the studies conducted in Sao Paulo, they are not directly comparable as Gouveia et al. [11] examined birth weight in relation to air pollution in a time series approach using mean exposures to citywide levels of air pollutants during each trimester of pregnancy. Medeiros et al. [24] studied only mothers residents in districts located in the southern region of the city, notably the poorest ones.

Controlled animal experiments have shown that exposure to air pollution can affect birth weight. In studies carried out in Sao Paulo [34],[35] investigated the effects of exposure to PM_2.5_ on reproductive function of mice using exposure chambers with filtered and non-filtered air. Significant changes in fetal capillary surface area and in the calibers of maternal blood spaces were observed. Fetal weight was significantly 20% higher among mice that received filtered air compared to the non-filtered group. Furthermore, fetal weight was influenced by both pre-gestational and gestational exposure, and a significant interaction between these two factors was observed.

The present study was based on a considerable number of births since all LBW children in the year of 2006 were included and a random sample of controls was drawn. The large number of subjects included warranted adequate statistical power for the analysis and reduced the probability of chance. It should be emphasized that the control selection of this study was carefully designed to avoid selection bias, since they were randomly drawn and matched by sex and month of birth to cases. In addition, both the process of geocoding addresses and estimating the traffic-based exposure was performed without knowing if the subjects were cases or controls. Losses of eligible subjects were small and due only to incompleteness of addresses in the original database precluding their geocoding. As compared to subjects, whose addresses were successfully geocoded, cases and controls that were not included in our study had higher proportion of mixed race and of less educated mothers. A possible explanation for this is the fact that in Brazil mixed race and less education are frequently associated with people from lower socioeconomic status that live in slums or ‘non-official addresses’.

This study was based on data obtained from a comprehensive birth certificate registry of an information system considered to be of high quality. It provided several covariates used to adjust the multivariate models. However, there are important known risk factors for a low birth weight baby such as maternal smoking status, maternal associated morbidity, pre-pregnancy weight, and weight gain among others, which were not available from this information system. Therefore, it might be that some confounding remained in our analysis, particularly regarding maternal smoking status which is one the most important predictors of low birth weight.

A study conducted in São Paulo estimated that nearly 20% of women are smokers and this prevalence is inversely correlated with education [36]. Therefore, we believe that adjusting the final models for maternal education may have, at least partially, eliminated confounding due to maternal smoking.

Traffic-related air pollution exposure levels were estimated at the home address and we were not able to know if some of these women might have moved during the pregnancy period or if they spent most of their time somewhere else. Nonetheless, a study of intra-urban residential mobility indicated that residents of the metropolitan area of São Paulo spend an average time of 14 years in the same address [37].

Additionally, according to another study also conducted in Sao Paulo with mothers of recent born babies or that have a perinatal death [24], more than 50% of them were housewives, unemployed, retired, or students, which means they might have spent most of the time during pregnancy at home. In this study the authors suggest that even for mothers who worked, it is likely that they might have stayed mostly at home during the final months of pregnancy, thus enhancing their exposure to the local traffic-related air pollutants. It should also be noted that in São Paulo people keep windows open throughout the year due to its mild climate. Therefore, part of the outdoor pollution from traffic exhaust penetrates indoors [24].

Other sources of imprecision in our estimate of exposure include the lack of information on wind patterns and local topography, which may alter the spatial distribution of air pollutants (e.g., traffic-related air pollution may be different for those living upwind or downwind) and the different types of vehicles’ fuel, such as gasoline, ethanol, or diesel vehicles, which have different emission profiles. These are all potential sources of variability in our estimates of exposure, but one does not expect them to be differentially distributed between cases and controls.

Another possible limitation of this study is the one-year difference between birth records (2006) and traffic data (2007). The counts and simulations of traffic are conducted by the Traffic Engineering Company in decennial periods thus the most recent and complete information available were from 2007 and birth records from 2007 were not available at the time we conducted this study. However, we believe this one-year lag did not introduce any noise in our analysis since changes in traffic volume and distribution were probably not substantial from one year to the other.

Additionally, exposure to traffic related air pollution was based on annual averages of traffic counts which might not reflect seasonal, monthly or other temporal differences in vehicle traffic. This data did not allow us to take into account the temporal variations in exposure (month or trimester of pregnancy) in order to increase the accuracy of our indicators. On the other hand, this study has the advantage of examining exposure with accurate spatial variability which is an important feature since air pollution levels vary considerably across different areas of a city, i.e. they are higher close to busy roads [23].

LUR modeling is an effective approach to assess the intraurban variability of air pollution [22]. However among the three indicators of exposure our LUR-PM_10_ model was probably the most inaccurate. LUR models require a sufficient network of sampling sites, properly selected to represent the gradient of exposure in the study population [38]. This condition is clearly not fulfilled in this study, where 09 monitoring stations were used as sites for LUR modeling. Moreover the fact that the final model only included one predictor variable (light traffic within 250 m) is also indicative of the poor performance of our LUR model.

This study found a decreased risk of LBW children associated with higher traffic related air pollution. At the same time we observed that mothers with higher exposure were also those with better SEP assessed by both indicators education and neighborhood level income. This pattern of exposure is the opposite observed in North America where studies indicate that people with lower SEP tend to live in areas with higher levels of air pollution [26],[27],[28].

Therefore, this unexpected association might be due to the fact that protection against LBW arising from a better SEP (by means of a better nutrition, antenatal health care access, etc.) is much stronger than the effect of exposure to air pollution. In other words, the effect of air pollution is not sufficient to increase the risk of LBW for these mothers.

In addition, it might also be that mothers with advantaged SEP, although residing in areas of higher vehicular traffic may not in fact be more exposed to air pollution. These areas probably have a greater number of new vehicles with lower emission factors compared to the periphery of the city. In addition, trucks and heavy duty vehicles are restricted to circulate in these central areas during most hours of the day. Finally, we can hypothesize that those mothers living in poorer neighborhoods can be more exposed to traffic-related air pollution not at home but when commuting to work.

Therefore, the strong and complex relationship between mothers’ SEP and exposure to traffic-related air pollution was not fully cleared in our models. Patterns of maternal exposure vary between individuals and covaried with SEP in different ways as seen in our analysis.

Several approaches have been proposed to more accurately determine population exposure to air pollution. However, the validity of these approaches has not always been examined [39]. The assessment of exposure to air pollution will continue to be a major issue in epidemiological studies, especially for places with large social disparities, as the case of São Paulo. Future studies with additional information about patterns of daily activity and lifestyle (e.g. car use) may help to solve this problem and provide better estimates of the effect of air pollution on pregnancy outcomes.

## Supporting Information

Table S1 **Adjusted odds ratios (AOR) for LBW for each covariate included in the adjusted final model for distance.**
(DOCX)

Table S2 **Adjusted odds ratios (AOR) for LBW for each covariate included in the adjusted final model for DWTD.**
(DOCX)

Table S3 **Adjusted odds ratios (AOR) for LBW for each covariate included in the adjusted final model for LUR-PM_10_.**
(DOCX)

Table S4 **Quartiles of the indicators of exposure and neighborhood-level income between cases of LBW and controls.**
(DOCX)

Table S5 **Quartiles of the indicators of exposure and maternal education between cases of LBW and controls.**
(DOCX)
