# Supplementary material for: Socioeconomic Position and Low Birth Weight among Mothers Exposed to Traffic-Related Air Pollution
Source: PLoS One. 2014 Nov 26;9(11):e113900. doi: 10.1371/journal.pone.0113900 (PMC4245223; doi:10.1371/journal.pone.0113900)
Supplement: Table S2 — Adjusted odds ratios (AOR) for LBW for each covariate included in the adjusted final model for DWTD. (DOCX) [file pone.0113900.s002.docx]

**Table S2.** Adjusted odds ratios (AOR) for LBW for each covariate included in the adjusted final model for DWTD.

| **DWTD (vehicles/hour)** | **AOR^a^ (95% CI)** | **p-value** |
| --- | --- | --- |
| <22.5 | *1.00* |  |
| 22.5 to <188.7 | *0.96 (0.86; 1.07)* | *<0.001* |
| 188.7 to <763.6 | *0.91 (0.82; 1.02)* |  |
| 763.6 to ≤10.331.1 | *0.90 (0.80; 1.01)* |  |
| **Antenatal care** |  |  |
| No visits | *2.57 (1.81; 3.65)* |  |
| 1 to 3 | *1.78 (1.49; 2.14)* | *<0.001* |
| 4 to 6 | *1.27 (1.15; 1.40)* |  |
| ≥ 7 | *1.00* |  |
| **Maternal education** |  |  |
| ≤ 3 years | *1.20 (0.97; 1.48)* |  |
| 4 to7 years | *1.32 (1.16; 1.52)* | *<0.001* |
| 8 to 12 years | *1.08 (0.97; 1.21)* |  |
| >12 years | *1.00* |  |
| **Number of previous births** |  |  |
| No child | *1.544 1.417 1.682* |  |
| 1 to 3 | *1.00* | *<0.001* |
| ≥4 | *1.00 (0.82; 1.21)* |  |
| **Marital status** |  |  |
| Single | *1.10 (1.01; 1.21)* |  |
| Married | *1.0* | *0.001* |
| Widow | *1.33 (0.59; 3.00)* |  |
| Separed/divorced | *1.24 (0.87; 1.77)* |  |
| Consensual union | *2.28 (1.52; 3.42)* |  |
| **Maternal age** |  |  |
| <20 | *1.07 (0.96; 1.21)* |  |
| 20 to 29 | *1.00* | *0.006* |
| 30 to 39 | *1.14 (1.04; 1.25)* |  |
| ≥40 | *1.34 (1.07; 1.68)* |  |
| **Number of previous stillbirths** |  |  |
| No child | *1.00* | *<0.001* |
| ≥1 | *1.35 (1.17; 1.57)* |  |
| **Delivery** |  |  |
| Vaginal | *1.00* | *0.226* |
| Cesarean | *1.05 (0.97; 1.14)* |  |
| **Neighborhood-level income^§^** |  |  |
| <3.35 | *1.09 (0.97; 1.23)* |  |
| 3.35 to <4.62 | *1.12 (1.00; 1.26)* | *0.234* |
| 4.62 to 7.16 | *1.04 (0.93; 1.16)* |  |
| ≥7.16 | *1.00* |  |
| ^§^quartiles of minimum wages |  |  |
