# Supplementary material for: Socioeconomic Position and Low Birth Weight among Mothers Exposed to Traffic-Related Air Pollution
Source: PLoS One. 2014 Nov 26;9(11):e113900. doi: 10.1371/journal.pone.0113900 (PMC4245223; doi:10.1371/journal.pone.0113900)
Supplement: Table S3 — Adjusted odds ratios (AOR) for LBW for each covariate included in the adjusted final model for LUR-PM10. (DOCX) [file pone.0113900.s003.docx]

**Table S3.** Adjusted odds ratios (AOR) for LBW for each covariate included in the adjusted final model for LUR-PM_10_.

| **LUR-PM_10_ (µg/m³)** | **AOR^a^ (95% CI)** | **p-value** |
| --- | --- | --- |
| <35.3 | *1.00* |  |
| 35.3 to <37.0 | *0.93 (0.83; 1.03)* | *0.044* |
| 37.0 to <40.4 | *0.88 (0.79; 0.98)* |  |
| 40.4 to ≤108.2 | *0.86 (0.76; 0.96)* |  |
| **Antenatal care** |  |  |
| No visits | *2.39 (1.70; 3.37)* |  |
| 1 to 3 | *1.80 (1.50; 2.16)* | *<0.001* |
| 4 to 6 | *1.26 (1.14; 1.39)* |  |
| ≥ 7 | *1.00* |  |
| **Maternal education** |  |  |
| ≤ 3 years | *1.19 (0.97; 1.47)* |  |
| 4 to7 years | *1.33 (1.16; 1.52)* | *<0.001* |
| 8 to 12 years | *1.08 (0.97; 1.21)* |  |
| >12 years | *1.00* |  |
| **Number of previous births** |  |  |
| No child | *1.54 (1.42; 1.68)* |  |
| 1 to 3 | *1.00* |  |
| ≥4 | *1.0 (0.82; 1.21)* |  |
| **Marital status** |  |  |
| Single | *1.11 (1.01;1.21)* |  |
| Married | *1.00* |  |
| Widow | *1.34 (0.59; 3.02)* | *0.001* |
| Separed/divorced | *1.23 (0.86; 1.76)* |  |
| Consensual union | *2.22 (1.49; 3.30)* |  |
| **Maternal age** |  |  |
| <20 | *1.07 (0.95; 1.20)* |  |
| 20 to 29 | *1.00* | *0.004* |
| 30 to 39 | *1.14 (1.04; 1.25)* |  |
| ≥40 | *1.36 (1.09; 1.71)* |  |
| **Number of previous stillbirths** |  |  |
| No child | *1.00* |  |
| ≥1 | *1.35 (1.16; 1.56)* | *<0.001* |
| **Delivery** |  |  |
| Vaginal | *1.00* |  |
| Cesarean | *1.06 (0.97; 1.15)* | *0.19* |
| **Neighborhood-level income^§^** |  |  |
| <3.35 | *1.06 (0.94; 1.20)* |  |
| 3.35 to <4.62 | *1.11 (0.98; 1.25)* | *0.332* |
| 4.62 to 7.16 | *1.03 (0.92; 1.15)* |  |
| ≥7.16 | *1.00* |  |
| ^§^quartiles of minimum wages |  |  |
