# Supplementary material for: Socioeconomic Position and Low Birth Weight among Mothers Exposed to Traffic-Related Air Pollution
Source: PLoS One. 2014 Nov 26;9(11):e113900. doi: 10.1371/journal.pone.0113900 (PMC4245223; doi:10.1371/journal.pone.0113900)
Supplement: Table S4 — Quartiles of the indicators of exposure and neighborhood-level income between cases of LBW and controls. (DOCX) [file pone.0113900.s004.docx]

**Table S4.** Quartiles of the indicators of exposure and neighborhood-level income between cases of LBW and controls.

|  | **Neighborhood-level income (quartiles of minimum wages)** | | | |
| --- | --- | --- | --- | --- |
| **Quartiles** | **<3.35** | **3.35 to <4.62** | **4.62 to 7.16** | **≥7.16** |
| **LUR-PM_10_ (µg/m³)^*^** |  |  |  |  |
| <35.3 | *1,318 (45.9%)* | *923 (31.7%)* | *516 (17.8%)* | *244 (8.4%)* |
| 35.3 to <37.0 | *840 (29.3%)* | *827(28.4%)* | *653 (22.6%)* | *466 (16.1%)* |
| 37.0 to <40.4 | *530 (18.5%)* | *702 (24.1%)* | *837 (28.9%)* | *828 (28.5%)* |
| 40.4 to ≤108.2 | *182 (6.3%)* | *460 (15.8%)* | *889 (30.7%)* | *1,365 (47.0%)* |
| **DWTD (vehicles/hour)^*^** |  |  |  |  |
| <22.5 | *1,188 (43.4%)* | *934 (32.3%)* | *511 (17.7%)* | *222 (7.6%)* |
| 22.5 to <188.7 | *752 (27.5%)* | *814 (28.2%)* | *765 (26.4%)* | *525 (18.1%)* |
| 188.7 to <763.6 | *488 (17.8%)* | *687 (23.8%)* | *846 (29.2%)* | *830 (28.6%)* |
| 763.6 to ≤10,331.1 | *307 (11.2%)* | *453 (15.7%)* | *771 (26.7%)* | *1,325 (45.7%)* |
| **Distance (meters)^*^** |  |  |  |  |
| <249.4 | *247 (8.6%)* | *497 (17.1%)* | *818 (28.3%)* | *1,333 (45.9%)* |
| 249.4 to <547.0 | *421 (14.7%)* | *591 (20.3%)* | *880 (30.4%)* | *1,003 (34.6%)* |
| 547.0 to <1,126.4 | *788 (27.5%)* | *835 (28.7%)* | *782 (27.0%)* | *489 (16.8%)* |
| 1,126.4 to ≤22,263.7 | *1,414 (49.3%)* | *989 (34.0%)* | *415 (14.3%)* | *78 (2.7%)* |
| *^*^*χ² test p ≤ 0.001 | | | | |
