# Supplementary material for: Socioeconomic Position and Low Birth Weight among Mothers Exposed to Traffic-Related Air Pollution
Source: PLoS One. 2014 Nov 26;9(11):e113900. doi: 10.1371/journal.pone.0113900 (PMC4245223; doi:10.1371/journal.pone.0113900)
Supplement: Table S5 — Quartiles of the indicators of exposure and maternal education between cases of LBW and controls. (DOCX) [file pone.0113900.s005.docx]

**Table S5.** Quartiles of the indicators of exposure and maternal education between cases of LBW and controls.

|  | **Maternal education (years)** | | | |
| --- | --- | --- | --- | --- |
| **Quartiles** | **0 - 3** | **4 - 7** | **8 - 11** | **≥ 12** |
| **LUR-PM_10_ (µg/m³)***^*^* |  |  |  |  |
| <35.3 | *176 (34.4%)* | *754 (30.0%)* | *1,686 (27.0%)* | *341 (15.7%)* |
| 35.3 to <37.0 | *110 (21.5%)* | *618 (24.6%)* | *1,579 (25.2%)* | *445 (20.5%)* |
| 37.0 to <40.4 | *106 (20.7%)* | *596 (23.7%)* | *1,535 (24.5%)* | *635 (29.3%)* |
| 40.4 to ≤108.2 | *119 (23.3%)* | *549 (21.8%)* | *1,455 (23.3%)* | *747 (34.5%)* |
| **DWTD (vehicles/hour)***^*^* |  |  |  |  |
| <22.5 | *160 (32.1%)* | *660 (26.8%)* | *1,670 (27.1%)* | *319 (14.8%)* |
| 22.5 to <188.7 | *116 (23.2%)* | *644 (26.1%)* | *1,572 (25.5%)* | *481 (22.3%)* |
| 188.7 to <763.6 | *113 (22.6%)* | *570 (23.1%)* | *1,523 (24.7%)* | *634 (29.4%)* |
| 763.6 to ≤10,331.1 | *110 (22.0%)* | *591 (24.0%)* | *1,402 (22.7%)* | *726 (33.6%)* |
| **Distance (meters)***^*^* |  |  |  |  |
| <249.4 | *119 (23.3%)* | *578 (23.0%)* | *1,422 (22.7%)* | *746 (34.4%)* |
| 249.4 to <547.0 | *95 (18.6%)* | *549 (21.8%)* | *1,536 (24.6%)* | *684 (31.5%)* |
| 547.0 to <1,126.4 | *132 (25.8%)* | *670 (26.6%)* | *1,625 (26.0%)* | *437 (20.2%)* |
| 1,126.4 to ≤22,263.7 | *65 (32.3%)* | *720 (28.6%)* | *1,672 (26.7%)* | *301 (13.9%)* |
| *^*^*χ² test p ≤ 0.001 | | | | |
